# *Crlz-1* Homozygous Null Knockout Mouse Embryos Are Lethally Stopped in Their Early Development

**DOI:** 10.3390/genes13030511

**Published:** 2022-03-14

**Authors:** Seung-Young Choi, Joo-Hyun Pi, So-Eun Jeong, Chang-Joong Kang

**Affiliations:** Department of Genetics and Biotechnology, College of Life Sciences, Kyung Hee University, 1732 Deogyeong-daero, Giheung, Yongin 17104, Korea; csy4343@naver.com (S.-Y.C.); pi0706@naver.com (J.-H.P.); oriquack@naver.com (S.-E.J.)

**Keywords:** *Crlz-1*, *Utp3*, *Sas10*, knockout mouse, early embryonic lethality

## Abstract

Although the conditional gene knockout (KO) is a better choice for observing its phenotype in a specific cell, tissue, and/or organ, the simple null gene KO could nevertheless be attempted initially to scan its overall phenotypes at the level of the whole-body system, especially for a new gene such as *Crlz-1*. Therefore, with a hope to glean phenotypic clues for *Crlz-1* at the whole-body system, we attempted to generate its null KO mice. Contrary to our original desire, *Crlz-1* homozygous null KO mice were not born. However, in the chasing of their homozygous KO embryos, they were found to be lethally impaired from early development, remaining in a state of small globular mass without ever leading to a body shape, indicating the critical role of *Crlz-1* as a Wnt target gene for the proliferation and/or differentiation of cells during early mouse embryonic development.

## 1. Introduction

*Crlz-1* (charged amino acid-rich leucine zipper-1), also called *Utp3* (U three protein 3) as is involved in the processing of rRNA by the small subunit processome (SSU) [1], was first reported as *Sas10* (something about silencing 10) with its protein’s transcriptional derepression activity in yeast [2], and was later cloned by its protein’s ability to bind CBFβ (core binding factor β) in the mice [3]. As its name implies, Crlz-1 with a sequence of 469 amino acids contains a high proportion of charged amino acids and might be expected to contain several functional domains, such as CBFβ binding and nuclear localization signal (NLS) motifs.

In our hands, *Crlz-1* was initially found to correspond to a novel pre-B cell-specific gene [4,5], which had been sought in the vicinity of *IgJ* gene locus on the mouse chromosome 5 because the same enhancer chromatin that was opened to express *IgJ* in the plasma cell stage [6] was also opened in the pre-B cell stage, despite the closed chromatin of *IgJ* gene locus and no *IgJ* expression, possibly to drive the pre-B cell-specific expression of a certain neighboring gene [7]. Notably, the promoter of *Crlz-1* turned out to be bound by LEF-1 (lymphoid enhancer factor-1) with β-catenin in pre-B cells, which led to the discovery that this gene is a target of the canonical Wnt (wingless-related MMTV integration site) signaling pathway [8,9]. Furthermore, *Crlz-1*, as induced by the Wnt/β-catenin signaling pathway, was found to play a critical role in the proliferating B cells such as pre-B [9] and germinal center (GC) centroblast cells [10]. Interestingly, it was also found to be expressed in the proliferating spermatogonia and Sertoli cells, as well as differentiating spermatids in the testis, indicating some roles during its development and/or spermatogenesis [11].

Crlz-1 was demonstrated to be localized within the nucleus [12], as anticipated by both its transcriptional derepression activity as Sas10 in yeast [2] and a constituent of SSU as Utp3 during rRNA processing [1]. Mechanistically, in accordance with its original cloning by CBFβ as a bait of yeast two-hybrid screen [3], Crlz-1 was demonstrated to work by mobilizing the cytoplasmic CBFβ into nucleus to allow its heterodimerization with Runx (runt-related transcription factor), and thereby to form a higher affinity form of Runx/CBFβ heterodimer in both pre-B and GC centroblast cells [9,10,12]. Consequently, in the case of pre-B cells, Runx/CBFβ turned on the expression of *VpreB* and *λ5* genes, leading to the assembly of pre-BCR (B cell receptor) and its signaling to proliferate pre-B cells for their clonal diversity during early B cell development [9], whereas, in the case of GC centroblast cells, Runx/CBFβ turned on the expression of the GC master *Bcl-6* gene, leading to the regulation of its many downstream target genes to proliferate centroblast cells without their differentiation into plasma cells during humoral immune GC reactions, which are essential for antibody affinity maturation, class switch recombination, and memory B cell generation [10].

In the present work, although we had hoped to glean some overall phenotypic clues for the physiological roles of *Crlz-1* by generating its homozygous null KO mice, they were not born despite genotyping 160 pups of 33 litters from the matings of *Crlz-1* heterozygous KO mice by themselves. However, when we chased for its homozygous KO embryos after sacrificing their pregnant mice at various days post coitum (dpc), we did finally detect *Crlz-1* homozygous KO embryos that were lethally impaired from their early development as compared to their wild-type and heterozygous KO littermates, potentially indicating the critical role of *Crlz-1* for the proliferation and/or differentiation of cells during embryogenesis.

## 2. Materials and Methods

### 2.1. Construction of Crlz-1 (Utp3) Null KO Mice

In order to generate *Crlz-1* (*Utp3*) null KO mice, Utp3^tm1(KOMP)Vlcg^ embryo stem cells (Utp3_AH7, UC Davis KOMP Repository) [13] were purchased and outsourced to a biotechnology company (Macrogen, Seoul, Korea) for the production service of its heterozygous KO founder mice (C57BL/6N strain). Two *Crlz-1* heterozygous KO founder female mice, which the service company supplied to us, were initially mated with our purchased wild-type C57BL/6N male mice (Koatech, Pyeongtaek, Korea) to increase their population and, thereafter, were bred by mating among their own different litters to maintain them as well as to screen for *Crlz-1* homozygous KO pups and embryos. All the mice experiments were performed with the approval (KHUASP(SU)-18-D) of Kyung Hee University Institutional Animal Care and Use Committee and thereby according to its guidelines.

### 2.2. Embryo Isolation

This experimental procedure was performed by following the protocols provided by two references [14,15]. The pregnant female mouse at various dpc as counted after seeing a vaginal plug was euthanized by CO_2_ asphyxiation, sprayed with 70% ethanol, and incised along the midline of the abdomen by pinching the skin. Subsequently, the peritoneum was cut to expose the abdominal cavity, in which the reproductive organs were located in the dorsal region of the cavity. The uterus was taken out by cutting below the oviducts as well as along the mesometrium, and placed in the ice-cold PBS. The uterus was then cut between its embryo implantation sites. In each of these uterine sections with its implanted embryo, the muscular uterus layer was peeled off to take the decidua tissue-enveloped embryo, which was then clipped off at its apex to remove a portion of the decidua tissue. Subsequently, the embryo with its Reichert’s membrane was shelled out by tearing apart its decidua tissue layer. Finally, under the anatomical microscope (S8APO, Leica, Wetzlar, Germany), the embryo was isolated by carefully removing its Reichert’s membrane as well as the ectoplacental trophoblast cone. Its mass or tail was clipped off and treated with 4 µL of proteinase K (10 mg/mL) in 196 µL of lysis buffer (0.1 M Tris-HCl, pH 8.0, 0.2 M NaCl, 5 mM EDTA, 0.4% SDS) at 55 °C overnight. The genomic DNA was extracted as usual and used for PCR.

### 2.3. Genotyping by PCR (Polymerase Chain Reaction)

**Figure 1:**
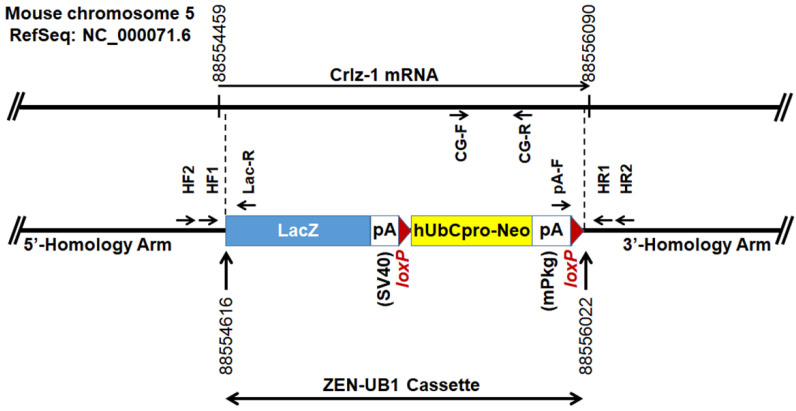
The map of ZEN-UB1 cassette-targeted *Crlz-1* KO locus is schematically drawn at the bottom, with that of wild-type *Crlz-1* locus aligned at the top, including the sites of eight arrowed primers for genotyping PCR. The numbers on the wild-type locus map at the top indicate the *Crlz-1* transcription start and stop sites, whereas the ones on the targeted locus map at the bottom indicate the *Crlz-1* deletion points. The numbers are based on the RefSeq of NC_000071.6 in the mouse chromosome 5.

Based on the *Crlz-1* KO construction map on the mouse chromosome 5 (Figure 1), which was informed by UC Davis KOMP Repository, eight primers, as indicated by arrows on the map, were designed to genotype KO pups and embryos by PCR, which was performed in essentially the same way as reported previously [9,10]. Two 5′-homology arm forward primers (HF1; ATA TCC TTG GCC TAG CGT TCC, HF2; AAC TTC CAC TCT GGC AAA CGG), which are paired with the LacZ reverse primer (Lac-R; GTC TGT CCT AGC TTC CTC ACT G) of the targeting reporter-selection cassette (ZEN-UB1, KOMP), generate the PCR product sizes of 448 and 671 bp, respectively. Two 3′-homology arm reverse primers (HR1; GGT TAG TTA TGC ACA GGC TCC, HR2; CCC CCA TGA CTG TCA AAG GTT), which are paired with the mPkg-pA forward primer (pA-F; TCA TTC TCA GTA TTG TTT TGC C) of the cassette, generate the PCR product sizes of 314 and 542 bp, respectively. The annealing temperature for the primer pairs of HF1 or HF2 with Lac-R was 64 °C, whereas that for the primer pairs of HR1 or HR2 with pA-F was 60 °C.

The KO homozygosity of ZEN-UB1 cassette-targeted *Crlz-1* locus was subsequently judged by the absence of *Crlz-1* gene band, despite the presence of *β-actin* gene band as an internal control, in the PCR performed using the same genomic DNA with the gene-specific primers, the sequences of which are as follows: *Crlz-1* gene forward (CG-F on the map; CAT CCT GTT ATA GAA AGG CTT GTT ACC), *Crlz-1* gene reverse (CG-R on the map; CTA TTT TTA GCA ATC TGG TAG GTA ATG), *β-actin* gene forward (TGA ACC CTA AGG CCA ACC GTG), and *β-actin* gene reverse (GCA GCT CAT AGC TCT TCT CCA GGG). Both of the gene-specific PCRs, which were performed using the same annealing temperature of 56 °C, generated their expected DNA sizes of 356 bp (*Crlz-1*) and 399 bp (*β-actin*), respectively.

### 2.4. In Situ Hybridization (ISH) and Reverse Transcription (RT)-PCR

These experiments were performed in essentially the same way as reported previously [10,11].

### 2.5. Image Analysis and Statistics

The embryo, ISH and RT-PCR images were analyzed by the NIH-provided freely available ImageJ software. Where necessary, the paired two-tailed Student’s *t* test was employed to obtain the *p* value, where *p* ≤ 0.05 was considered to be statistically significant, and the standard error of the mean (SEM) was calculated using Excel.

## 3. Results

### 3.1. Crlz-1 Homozygous Null KO Mouse Embryos Are Lethally Impaired in Their Development, and So Their Live Pups Are Not Born

**Figure 2:**
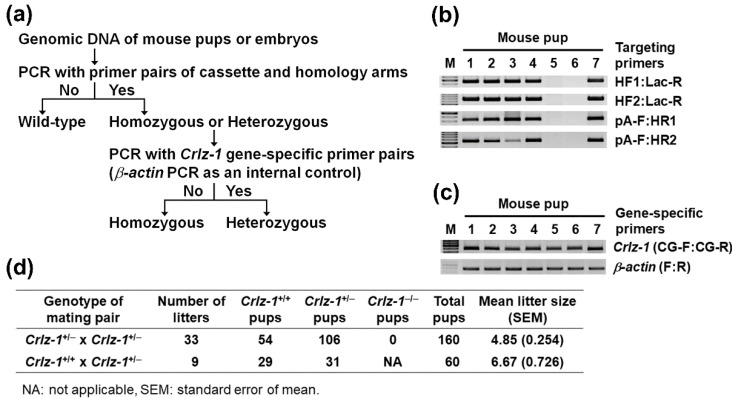
*Crlz-1*^−/−^ homozygous null KO pups were not born from the mating pairs of *Crlz-1*^+/−^ heterozygous KO mice by themselves. (**a**) A scheme of PCR genotyping strategy is drawn. (**b**) PCR using the genomic DNA extracted from the tails of pups was performed with the primers indicated over the cassette-targeted *Crlz-1* KO locus in Figure 1 to analyze whether the *Crlz-1* locus was targeted by the ZEN-UB1 cassette, although it could not still be said which embryos were heterozygously or homozygously targeted. The representative genotyping PCR results from a litter are shown, where #1–4 and #7 pups are found to be targeted, but #5–6 pups are wild-type. (**c**) PCR using the same genomic DNA as in panel b was performed with the primer pairs specific for *Crlz-1* and *β-actin* genes to determine which pups were homozygously targeted, as judged by the absence of *Crlz-1* gene despite the presence of *β-actin* gene as an internal control. The representative genotyping PCR results from the same litter as in panel b are shown, where all the targeted pups, i.e., #1–4 and #7 pups, are found to be heterozygous. (**d**) The genotyping results of 160 pups from 33 litters born in the matings of *Crlz-1*^+/−^ heterozygous KO mice by themselves, as analyzed in panels b and c, are summarized in the top row of the table. Those of 60 pups from 9 litters born from the matings between *Crlz-1*^+/+^ wild-type and *Crlz-1*^+/−^ heterozygous mice are also included in the bottom row as a control. M: DNA size marker.

In order to analyze the function of *Crlz-1* at the whole-organism level, we first planned to generate *Crlz-1* null knockout (KO) mice whose construction map is given in Figure 1. Contrary to our original desire, *Crlz-1* homozygous null KO mice were not born despite genotyping 160 pups of 33 litters from the matings between its heterozygous KO (*Crlz-1*^+/−^) mice by themselves, where wild-type (*Crlz-1*^+/+^) and heterozygous KO (*Crlz-1*^+/−^) pups of both sexes were born normally (Figure 2d, top row). The births of 54 wild-type (*Crlz-1*^+/+^) pups and 106 heterozygous KO (*Crlz-1*^+/−^) pups with no birth at all of their homozygous KO (*Crlz-1*^−/−^) pups from such matings corresponded to the mean litter size of 4.85 (Figure 2d, top row). These results were close to the theoretical 1:2 birth ratio of wild-type to heterozygous pups according to the Mendelian law, with about a 25% drop in the litter size due to no birth of its homozygous pups, as compared to the normal size of 6.5 for C57BL/6N mouse strain, as the supplier of its wild-type mice informed us. Our genotyping results from such matings of heterozygous mice by themselves were further supported by the fact that the control matings between *Crlz-1* wild-type (*Crlz-1*^+/+^) and its heterozygous KO (*Crlz-1*^+/−^) mice, where a total of 9 litters of 60 pups were obtained, gave birth to 29 wild-type and 31 heterozygous KO pups, satisfying the theoretical 1:1 Mendelian birth ratio of pup’s genotypes, with a roughly normal mean litter size of 6.67 (Figure 2d, bottom row).

**Figure 3:**
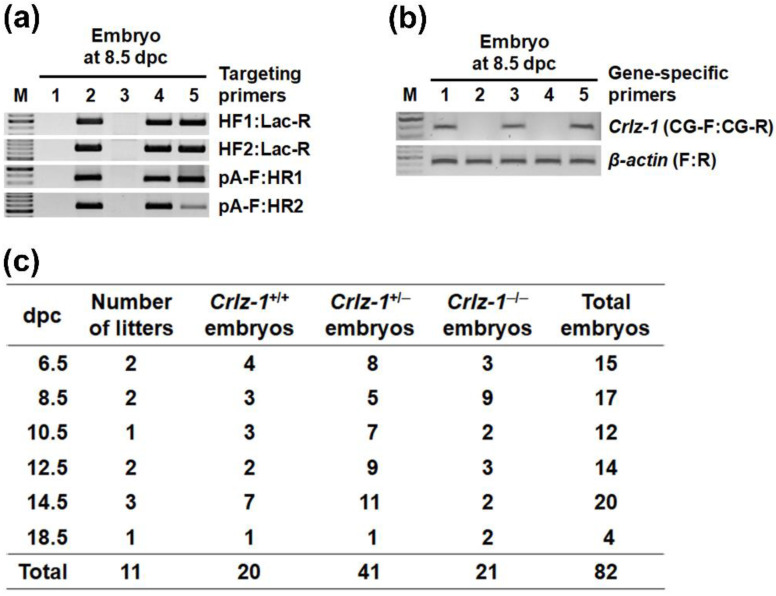
The embryos of various dpc from the mating pairs of *Crlz-1*^+/−^ heterozygous KO mice by themselves were chased for their *Crlz-1*^−/−^ homozygous KO genotypes by the same strategy as drawn in panel a of Figure 2. (**a**) PCR using the genomic DNA obtained from the embryos at various dpc was performed with the primer pair indicated on the right of each gel. The representative genotyping PCR results from an embryo litter of 8.5 dpc are shown, where #1 and #3 embryos are wild-type whereas #2 and #4–5 embryos are targeted. (**b**) PCR using the same genomic DNA was subsequently performed with the primer pairs specific for *Crlz-1* and *β-actin* genes to determine which embryos were homozygously targeted. The representative genotyping PCR results for the same litter in panel a are shown, where among the three targeted embryos, #2 and #4 embryos are found to be homozygous whereas #5 is heterozygous. (**c**) The embryos of each genotype at the various dpc are summarized in a table. M: DNA size marker.

**Figure 4:**
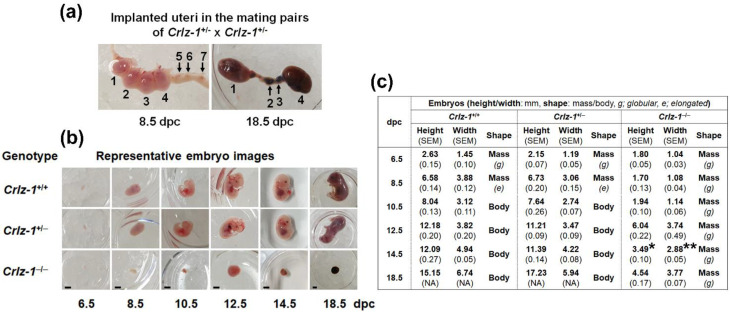
*Crlz-1*^−/−^ homozygous null KO mouse embryos were found to be lethally impaired in their development from around 8.5 dpc and evidently so at the later dpc. (**a**) The 8.5 (10× magnification) and 18.5 (4×) dpc images of uteri implanted with embryos of all three different genotypes from the mating pairs of heterozygous KO mice by themselves are shown. The implanted embryos are numbered for matching them to their genotypes as follows: for 8.5 dpc—*Crlz-1*^+/+^ (#2), *Crlz-1*^+/−^ (#1, 3, 4), and *Crlz-1*^−/−^ (#5, 6, 7); and for 18.5 dpc—*Crlz-1*^+/+^ (#4), *Crlz-1*^+/−^ (#1), and *Crlz-1*^−/−^ (#2, 3). (**b**) The representative images of *Crlz-1*^+/+^ wild-type, *Crlz-1*^+/−^ heterozygous KO and *Crlz-1*^−/−^ homozygous KO embryos at the various dpc are shown. In the normal control case of wild-type mice, the embryos at the early stages of 6.5–8.5 dpc were just a globular or an elongated mass of different sizes (observed with 20×), whereas the embryos at the late stages of 10.5–14.5 (10×) and 18.5 dpc (4×) had more or less a body shape. Scale bars: 2 mm. (**c**) The embryos of *Crlz-1* wild-type, heterozygous, and homozygous KO mice at various dpc are summarized in a table in terms of height, width, and shape, describing them as a globular or elongated mass and/or a body shape. Height: distance between brain top and hip bottom; width: distance between dorsal and ventral sides. At 14.5 dpc, * *p* ≤ 2.2 × 10^−10^ for homozygous versus wild-type and * *p* ≤ 3.5 × 10^−16^ for homozygous versus heterozygous; ** *p* ≤ 9.2 × 10^−12^ for homozygous versus wild-type and ** *p* ≤ 1.7 × 10^−7^ for homozygous versus heterozygous. NA: not applicable, SEM: standard error of the mean.

However, in the chasing of their *Crlz-1*^−/−^ homozygous KO embryos after sacrificing the pregnant mice from the matings of *Crlz-1*^+/−^ heterozygous KO mice by themselves at the various dpc, *Crlz-1*^−/−^ homozygous KO embryos were detected within their uteri (Figure 3 and Figure 4), although their development was observed to be stopped from around 8.5 dpc without ever leading to a body shape at the later dpc as compared to the normally developed ones of their *Crlz-1*^+/+^ wild-type or *Crlz-1*^+/−^ heterozygous littermates (Figure 4b), indicating that *Crlz-1* should play an essential role in the proliferation and differentiation of cells during embryogenesis. Experimentally, although there was a variation of 1–3 litter numbers analyzed at each dpc, a total sum of 82 embryos from the 11 litters chased from 6.5 to 18.5 dpc have nicely followed the theoretical Mendelian ratio of 1:2:1 among 20 wild-type, 41 heterozygous, and 21 homozygous embryos (Figure 3c), indicating that 11 litters with 82 embryos through the dpc might be a statistically justifiable number of litters to support the embryonic lethality of *Crlz-1* homozygous null KO mice.

### 3.2. The Development of Crlz-1^−/−^ Homozygous Null KO Mouse Embryos Was Stopped from Around 8.5 dpc, Remained in a State of Small Globular Mass, and Did Not Ever Lead to a Body Shape

Initially, *Crlz-1*^−/−^ homozygous null KO embryos were missed due to their early developmental stoppage until they were caught, owing to their similarly developed sizes as the *Crlz-1*^+/+^ wild-type or *Crlz-1*^+/−^ heterozygous littermates at 6.5 dpc (Figure 4b). Thereafter, when their embryos were cautiously searched along the implanted uteri, they could also be observed in a state of small globular mass at the later dpc (Figure 4a,b). To our surprise, throughout all the dpc, the *Crlz-1*^−/−^ homozygous null KO embryos were neither elongated nor shaped to a developmental body structure consisting of heart with vasculogenesis, head with rudimentary eyes, limb buds, and/or tail, which were clearly observed in the *Crlz-1*^+/+^ wild-type or *Crlz-1*^+/−^ heterozygous littermates (Figure 4b), indicating that Crlz-1 plays an essential role during the embryonic development of a body structure. Specifically, the development of the *Crlz-1* homozygous KO embryos started to differ sharply in terms of size and shape from 8.5 dpc as compared to the normally developing ones of their wild-type and heterozygous KO littermates from the same uterus (Figure 4a,b). The normal mouse embryos at 8.5 dpc might have just passed the gastrulating embryo stage with their developing germ layers in the post-implantation development, as depicted in the recent SnapShot presentation of Stem Cell Reports [16].

### 3.3. Crlz-1 Heterozygous KO Mice Looked Generally Normal despite of Its Haploinsufficient Expression

**Figure 5:**
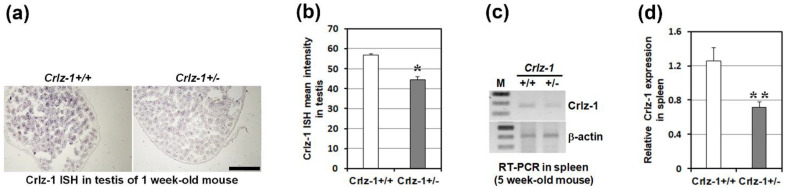
*Crlz-1* was expressed haploinsufficiently in its heterozygous KO mice. (**a**,**c**) The haploinsufficient expression of *Crlz-1* in its heterozygous KO mouse (*Crlz-1*^+/−^) was demonstrated in 1-week-old mouse testis by ISH in panel a, as well as in 5-week-old mouse spleen by RT-PCR in panel c, as compared to its expression in the wild-type littermate (*Crlz-1*^+/+^). (**b**,**d**) The ISH signal intensities from four representative tissue sections of each genotyped mouse testis as in panel a, and the RT-PCR signal intensities from four wild-type and three heterozygous spleens as in panel c, were statistically processed using the ImageJ program to obtain bar graphs with error bars of SEM in panels b and d, respectively. M: DNA size marker. Scale bar in panel a: 1 mm. * *p* ≤ 0.0005, ** *p* ≤ 0.034.

Testis and spleen, which are known to specifically express *Crlz-1* [11], were employed to check the haploinsufficient expression of *Crlz-1* in the heterozygous KO mice as compared to their wild-type littermates. Experimentally, due to the unavailability of antibodies specifically recognizing the mouse Crlz-1 protein, we opted to check the level of its mRNA expression, and did perform the in situ hybridization (ISH) in testis and RT-PCR in the spleen. In these experiments, a haploinsufficient expression of Crlz-1 mRNA in the heterozygous KO mice was certainly demonstrated as compared to the wild-type littermate (Figure 5), confirming our genotyping genomic DNA PCR results that one of two *Crlz-1* alleles was absent in these mice, at the level of mRNA expression. Despite a haploinsufficient expression of *Crlz-1*, the heterozygous KO mice looked generally normal as compared to their wild-type littermates throughout the experiments, although the breeding rate in the mating pairs of the heterozygous mice by themselves appeared to be slow (see Section 4).

## 4. Discussion

The embryonic lethality of *Crlz-1* homozygous null KO mice, and thus the failure of obtaining their pups, requires that its conditional KO mice should be pursued in the future to define its physiological functions in any specific cells, tissues, and/or organs during late embryonic development as well as after birth. The embryonic lethality could be explained by considering Wnt-targeted *Crlz-1* expression regulation and its protein function in human and/or mouse cells, as reported in our previous papers [8,9,10,12], as well as the transcriptional derepression activity of Sas10 as a Crlz-1 homologue in yeast [2] and the role of Crlz-1 as Utp-3 component of the rRNA-processing SSU complex, as reported by other researchers [1,17,18]. All of these potential explanations might not be mutually exclusive and together could be responsible for the developmentally stopped embryonic lethal phenotype of *Crlz-1* homozygous null KO mice.

We have shown that the Wnt signal induced the expression of Crlz-1, which was then demonstrated to mobilize the cytoplasmic CBFβ into the nucleus to form a higher affinity Runx/CBFβ heterodimer. Sequentially, Runx/CBFβ was shown to bind to its target regulatory chromatin loci to open the chromatin, and thus to express their corresponding genes, ultimately leading to the expression of cyclins D, G1 checkpoint regulators, for the rapid proliferation of cells. This working mechanism of Crlz-1 might partially explain the early developmentally stopped lethal phenotype of embryos in the *Crlz-1* homozygous KO mice, because the Wnt signal is reported to be involved in the proliferation and/or differentiation of cells during embryogenesis [19,20,21,22], and Runx/CBFβ is known to be essential for the early embryonic hematopoiesis [23,24,25] and osteogenesis [26]. The transcriptional derepression activity of Sas10 on the silenced chromatin, which was demonstrated by its overexpression in *Saccharomyces cerevisiae* [2], might also potentially be explained in a way similar to the molecular working mechanism of Crlz-1 as described above in mouse and/or human cells.

Another potential reason for the embryonic lethality might be related to the role of Crlz-1 as Utp-3 component of small subunit processome (SSU) during the rRNA processing. However, Utp-3 does not appear to be constitutively required as a house-keeping protein for the rRNA processing, as judged by the fact that it is highly expressed mainly in rapidly proliferating cells such as pre-B and GC centroblast cells [9,10], but not ubiquitously in all cells. Based on this information, it is speculated that Crlz-1 should be highly expressed in the proliferating embryonic cells to accelerate the supply of more translating ribosomes by participating as Utp-3 component of SSU, thereby speeding up the timely production of necessary amounts of proteins to match their rapid proliferation. The essential role of Crlz-1 as Utp-3 component of SSU during embryogenesis is further supported by recent reports that the production of seeds in *Arabidopsis thaliana* is impaired by aberrant rRNA expression and processing in the *THAL* (*Crlz-1* homologue) KO plant [17], and that its loss-of-function mutations result in an incomplete organogenesis during zebrafish embryogenesis due to the defects in rRNA processing [18], suggesting that Crlz-1 as Utp-3 component of SSU is critical during embryogenesis in both Arabidopsis and zebrafish as well.

Interestingly, although *Crlz-1* heterozygous KO mice looked generally normal, the time required to get a successful pregnancy from the mating pairs of its heterozygous KO mice by themselves was delayed, even no pregnancy at all in a few mating pairs; hence, it took longer to screen for the *Crlz-1* homozygous KO pups as well as to see their vaginal plugs for chasing their embryos. At present, although the reasons for the lower mating or pregnancy rate and thereby the slow breeding rate of *Crlz-1* heterozygous KO mice by themselves are not clear, one reason might be partially related to our previous report that Crlz-1 is prominently expressed in spermatogonia and Sertoli cells during early testis development and in spermatids during late spermatogenesis [11], indicating its potential roles for the generation and function of these reproductive cells in testis. However, contradicting this explanation, their reproductive organs regardless of their genders appeared to function normally, as judged by the fact that the wild-type and heterozygous KO embryos, both of which were born normally (Figure 2d, top row), were found together with the developmentally stopped smaller homozygous KO embryos within the same uterus (Figure 4a,b) in a roughly normal average litter size as well as approximately normal Mendelian genotypic ratio (Figure 3c). A further complication is that the low breeding rate did not happen when the heterozygous KO mouse was mated with the wild-type mouse (Figure 2d, bottom row).

## Data Availability

Not applicable.

## References

[1] Bernstein K.A., Baserga S.J. (2004). The small subunit processome is required for cell cycle progression at G1. Mol. Biol. Cell.

[2] Kamakaka R.T., Rine J. (1998). Sir- and silencer-independent disruption of silencing in Saccharomyces by Sas10p. Genetics.

[3] Sakuma T., Li Q.L., Jin Y., Choi L.W., Kim E.G., Ito K., Ito Y., Nomura S., Bae S.C. (2001). Cloning and expression pattern of a novel PEBP2β-binding protein (charged amino acid rich leucine zipper-1 [Crl-1]) in the mouse. Mech. Dev..

[4] Lim J.H., Cho S.J., Park S.K., Kim J., Cho D., Lee W.J., Kang C.J. (2006). Stage-specific expression of two neighboring *Crlz1* and *IgJ* genes during B cell development is regulated by their chromatin accessibility and histone acetylation. J. Immunol..

[5] Kim J.Y., Park S.K., Kim H.G., Cho S.J., Kim J., Kang C.J. (2006). The HSS3/4 enhancer of Crlz1-IgJ locus is another target of EBF in the pre-B cell stage of B cell development. Immunol. Lett..

[6] Kang C.J., Sheridan C., Koshland M.E. (1998). A stage-specific enhancer of immunoglobulin J chain gene is induced by interleukin-2 in a presecretor B cell stage. Immunity.

[7] Kang C.J., Oh U., Koshland M.E. (2000). Dynamic chromatin remodeling in the vicinity of J chain gene for the regulation of two stage-specific genes during B cell differentiation. Mol. Cells.

[8] Park S.K., Son Y., Kang C.J. (2011). A strong promoter activity of pre-B cell stage-specific Crlz1 gene is caused by one distal LEF-1 and multiple proximal Ets sites. Mol. Cells.

[9] Choi S.Y., Park S.K., Yoo H.W., Pi J.H., Kang C.J. (2016). Charged amino acid-rich leucine zipper-1 (Crlz-1) as a target of Wnt signaling pathway controls pre-B cell proliferation by affecting Runx/CBFβ-targeted *VpreB* and *λ5* genes. J. Biol. Chem..

[10] Choi S.Y., Pi J.H., Park S., Kang C.J. (2019). *Crlz-1* controls germinal center reaction by relaying a Wnt signal to the *Bcl-6* expression in centroblasts during humoral immune responses. J. Immunol..

[11] Lim J.H., Choi S.Y., Yoo H.W., Cho S.J., Son Y., Kang C.J. (2013). Crlz-1 is prominently expressed in spermatogonia and Sertoli cells during early testis development and in spermatids during late spermatogenesis. J. Histochem. Cytochem..

[12] Park S.K., Lim J.H., Kang C.J. (2009). Crlz1 activates transcription by mobilizing cytoplasmic CBFβ into the nucleus. Biochim. Biophys. Acta-Gene Regul. Mech..

[13] Valenzuela D.M., Murphy A.J., Frendewey D., Gale N.W., Economides A.N., Auerbach W., Poueymirou W.T., Adams N.C., Rojas J., Yasenchak J. (2003). High-throughput engineering of the mouse genome coupled with high-resolution expression analysis. Nat. Biotechnol..

[14] Shea K., Geijsen N. (2007). Dissection of 6.5 dpc mouse embryos. JoVE.

[15] Pryor S.E., Massa V., Savery D., Greene N.D.E., Copp A.J., Turksen K. (2012). Convergent extension analysis in mouse whole embryo culture. Planar Cell Polarity: Methods in Molecular Biology (Methods and Protocols).

[16] Rivron N., Fu J. (2021). SnapShot: Embryo models. Stem Cell Rep..

[17] Chen Y.J.C., Wang H.J., Jauh G.Y. (2016). Dual role of a SAS10/C1D family protein in ribosomal RNA gene expression and processing is essential for reproduction in *Arabidopsis thaliana*. PLoS Genet..

[18] Zhao S., Chen Y., Chen F., Huang D., Shi H., Lo L.J., Chen J., Peng J. (2019). Sas10 controls ribosome biogenesis by stabilizing Mpp10 and delivering the Mpp10–Imp3–Imp4 complex to nucleolus. Nucleic Acids Res..

[19] Xu Z., Robitaille A.M., Berndt J.D., Davidson K.C., Fischer K.A., Mathieu J., Potter J.C., Ruohola-Baker H., Moon R.T. (2016). Wnt/β-catenin signaling promotes self-renewal and inhibits the primed state transition in naïve human embryonic stem cells. Proc. Natl. Acad. Sci. USA.

[20] Lien W., Fuchs E. (2014). Wnt some lose some: Transcriptional governance of stem cells by Wnt/ß-catenin signaling. Genes Dev..

[21] Kurek D., Neagu A., Tastemel M., Tuysuz N., Lehmann J., van de Werken H.J.G., Philipsen S., van der Linden R., Maas A., van IJcken W.F.J. (2015). Endogenous WNT signals mediate BMP-induced and spontaneous differentiation of epiblast stem cells and human embryonic stem cells. Stem Cell Rep..

[22] Zhu D., Gong X., Miao L., Fang J., Zhang J. (2017). Efficient induction of syncytiotrophoblast layer II cells from trophoblast stem cells by canonical Wnt signaling activation. Stem Cell Rep..

[23] de Bruijn M., Dzierzak E. (2017). Runx transcription factors in the development and function of the definitive hematopoietic system. Blood.

[24] Sood R., Kamikubo Y., Liu P. (2017). Role of RUNX1 in hematological malignancies. Blood.

[25] Tober J., Yzaguirre A.D., Piwarzyk E., Speck N.A. (2013). Distinct temporal requirements for Runx1 in hematopoietic progenitors and stem cells. Development.

[26] Wu M., Wang Y., Shao J., Wang J., Chen W., Li Y. (2017). Cbfβ governs osteoblast−adipocyte lineage commitment through enhancing β-catenin signaling and suppressing adipogenesis gene expression. Proc. Natl. Acad. Sci. USA.

